# A new species of *Atractides* Koch, 1837 (Acari, Hydrachnidia, Hygrobatidae) from Ethiopia, with a discussion on the biodiversity of the genus *Atractides* in the Afrotropical region

**DOI:** 10.3897/zookeys.86.972

**Published:** 2011-03-19

**Authors:** Vladimir Pešić, Harry Smit

**Affiliations:** 1 Department of Biology, University of Montenegro, Cetinjski put b.b., 81000 Podgorica, Serbia and Montenegro; 2 Zoological Museum, University of Amsterdam, Plantage Middenlaan 64, 1018 DH Amsterdam, The Netherlands

**Keywords:** Water mites, taxonomy, new species, Africa, biodiversity

## Abstract

A new species of *Atractides* Koch, 1837 (Acari, Hydrachnidia) is described from Ethiopia. The world number of *Atractides* now tallies 297 species. The diversity of the genus *Atractides* in the Afrotropical region is briefly discussed.

## Introduction

Water mites of the genus *Atractides* Koch, 1837 have been found in all biogeographical regions except Australia and Antarctica. Gerecke (2003) reported 123 species from Europe, 72 from Asia, 27 from North America, 33 from Central and South America and 54 from Africa. Since then, many new species have been described (Pešić et al. 2004, Pešić and Smit 2009, Tuzovskij 2011), suggesting that many more remain to be discovered.

The aim of our paper is to describe a new *Atractides* species from Ethiopia. Additionally, the worldwide biodiversity of the genus *Atractides* Koch, 1837 of the world, with an emphasis on the Afrotropical region, is briefly discussed.

## Material and methods

Water mites were collected by hand netting, sorted on the spot from the living material, preserved in Koenike fluid and dissected as described elsewhere (e.g. Davids et al. 2007). Holotype and paratypes are deposited in the Zoological Museum in Amsterdam (ZMAN). All material has been collected by the junior author.

All measurements are given in μm. For a detailed description and discussion of the characteristics of the genus *Atractides* and a detailed methodological introduction, see Gerecke (2003).

The following abbreviations are used: Ac-1 = first acetabulum, alt. = altitude, asl. = above sea level, Cx-I = first coxae, dL = dorsal length, H = height, HB = central height, L = length, I-L-6 = Leg 1, sixth segment (tarsus), lL = lateral length, mL = medial length, P-1 = palp, first segment, S-1 = large proximal ventral seta at I-L-5, S-2 = large distal ventral seta at I-L-5, Vgl = ventroglandulare, vL = ventral length, W = width.

## Systematics

### Atractides (Atractides) ethiopiensis sp. n.

urn:lsid:zoobank.org:act:E968E96B-BAF1-4641-8DB4-3490FC07C28C

Figs 1 [Fig F2] 3 

#### Type series.

Holotype, male (ZMAN), dissected and slide mounted, Ethiopia, Roby River, 21.x.2006, 9°44.996N, 38°59.743E, 2507 m a.s.l. Paratypes: two males, one female (ZMAN, one female dissected and slide mounted), same data as holotype.

#### Diagnosis.

Dorsal integument striated, palp slender with P-2 straight in the both sexes, S-1 in female ending in a fine hair-like tip, excretory pore surrounded by distinct oval sclerite, Vgl-1 fused to Vgl-2.

#### Description.

**General features**. Dorsal integument: striated, muscle attachment plates unsclerotized. Coxal field: extended secondary sclerotization, caudal margin Cx-I broadly convex. Genital field: Ac in a weakly curved line. Excretory pore: sclerotized; Vgl-1 fused to Vgl-2. Palp: ventral margin of P-2 and ventral margin of P-3 slightly concave, ventral margin of P-4 straight, sword seta between ventral hairs, nearer to the distoventral hairs. Legs: I-L-5 S-1 and S-2 pointed, interspaced, S-2 basally enlarged, bluntly pointed; I-L-6 slender, curved, with maximum H proximally; leg claws with dorsal and ventral clawlets (Fig. 2C).

#### Morphology.

##### Male:

###### Idiosoma

**Figure 1.**
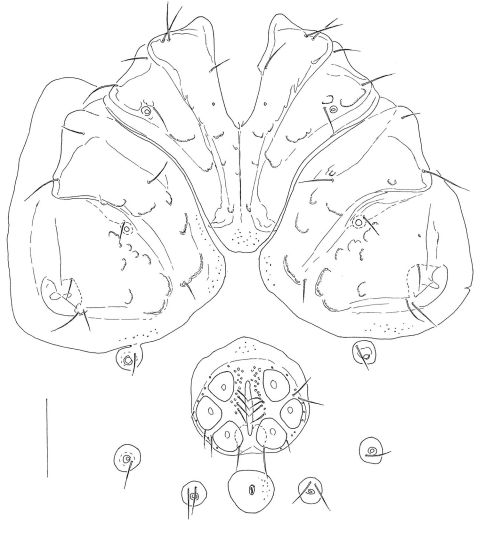
*Atractides ethiopiensis* sp. n., male: idiosoma, ventral view. Scale bar = 100 μm.

L/W 669-684/541-556; coxal field L 420; Cx-III W 484; Cx-I+II mL 156; lL Cx-I+II 265. Genital field (Fig. 1): anterior margin convex, with a border of secondary sclerotization convexely protruding, anterior margins of gonopore and Ac-1 considerably distant from anterior margin of genital field; L/W 150/150; L Ac 1–3: 38–41, 39–45, 39–41.

###### Palp

**Figure 2. F2:**
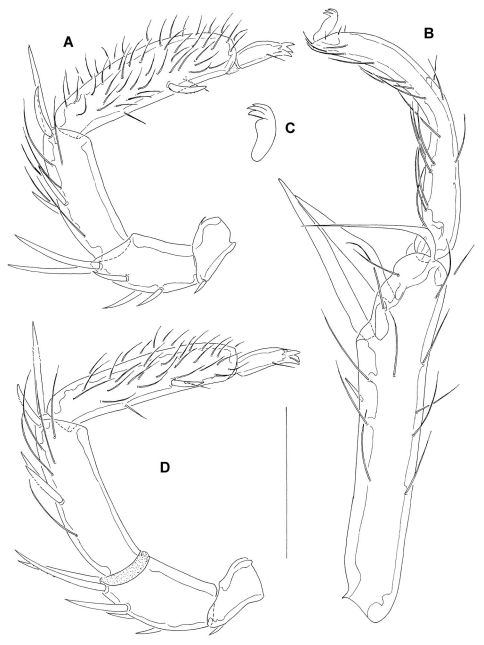
*Atractides ethiopiensis* sp. n., (**A–C** male, **D** female) **A, D** palp, medial view **B** I-L-5 and -6 **C** leg claw. Scale bar = 100 μm.

(Fig. 2A): total L 369, dL: P-1, 35; P-2, 72; P-3, 92; P-4, 131; P-5, 39; L ratio P-2/P-4, 0.55; P-4 club-shaped, with maximum H near distoventral hair.

I-L: I-L-5 dL 243, vL 169, dL/vL ratio 1.44, HB 50, dL/HB 4.86, S-1 L 121, L/W 11.2, S-2 L 95, L/W 5.6, distance S-1-2 23, L ratio S-1/2 1.27; I-L-6 L 166, HB 16, L/HB ratio 10.3; L ratio I-L-5/6 1.46.

##### Female.

###### Idiosoma

**Figure 3.**
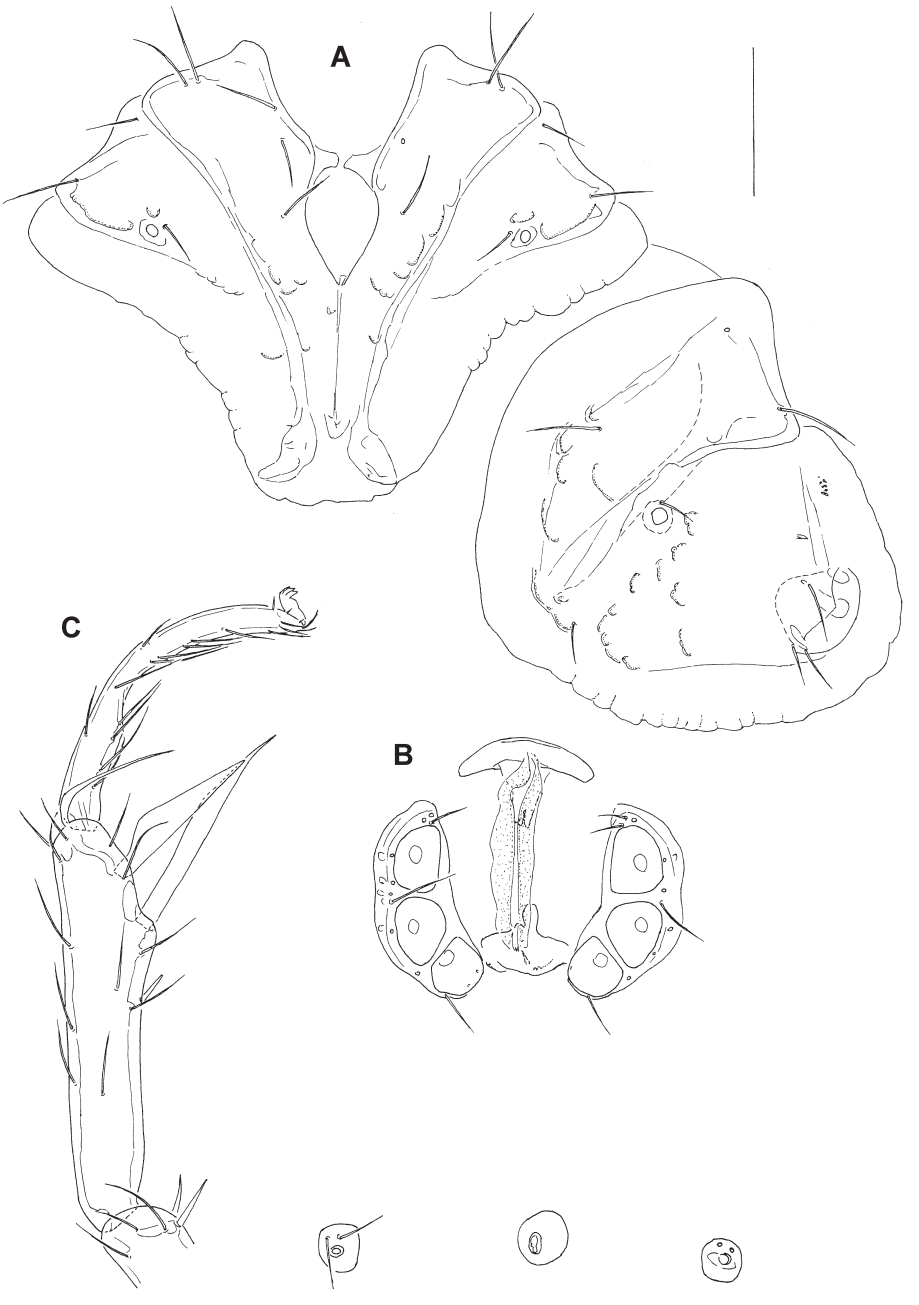
*Atractides ethiopiensis* sp. n., female **A** = coxal field **B** = genital field, excretory pore and Vgl-1 and -2 **C** = I-L-5 and -6. Scale bar = 100 μm.

L/W 1106/928; coxal field L 473; Cx-III W 644; Cx-I+II mL 147; lL Cx-I+II 284. Genital field (Fig. 3B): L/W 181/209; genital plate bean-shaped, with slightly concave medial margins, L 139; L Ac 1–3: 44, 47, 43.

###### Palp

(Fig. 2D): total L 417, dL: P-1, 48; P-2, 80; P-3, 115; P-4, 132; P-5, 42; L ratio P-2/P-4, 0.6; P-4 not club-shaped, with maximum H near proximoventral hair; chelicera total L 250, claw L 83.

I-L: I-L-5 dL 279, vL 186, dL/vL ratio 1.5, HB 65, dL/HB 4.3, S-1 ending in a fine hair-like tip (Fig. 3C) L 162, L/W 11.3, S-2 L 117, L/W 4.8, distance S-1-2 29, L ratio S-1/2 1.38; I-L-6 L 210, HB 19, L/HB ratio 11.1; L ratio I-L-5/6 1.33.

#### Etymology.

Named after its occurrence in Ethiopia.

#### Remarks.

Due to the striated integument, a slender palp with P-2 lacking ventrodistal projection and similar morphology of I-L-5 and -6, with S-1 in female ending in a fine hair-like tip *Atractides ethiopiensis* sp. n. resembles *Atractides latisetus* (K. Viets, 1916), a species known from Cameroon (K. Viets 1916), Liberia (Cook 1966), East and South Africa (Lundblad 1952, Viets 1964). This species can be easily distinguished from *Atractides ethiopiensis* sp. n., by a smooth excretory pore and unfused Vgl-1 and -2.

#### Distribution.

Ethiopia.

## Discussion

### a) The present biodiversity of Atractides

**Figure 4.**
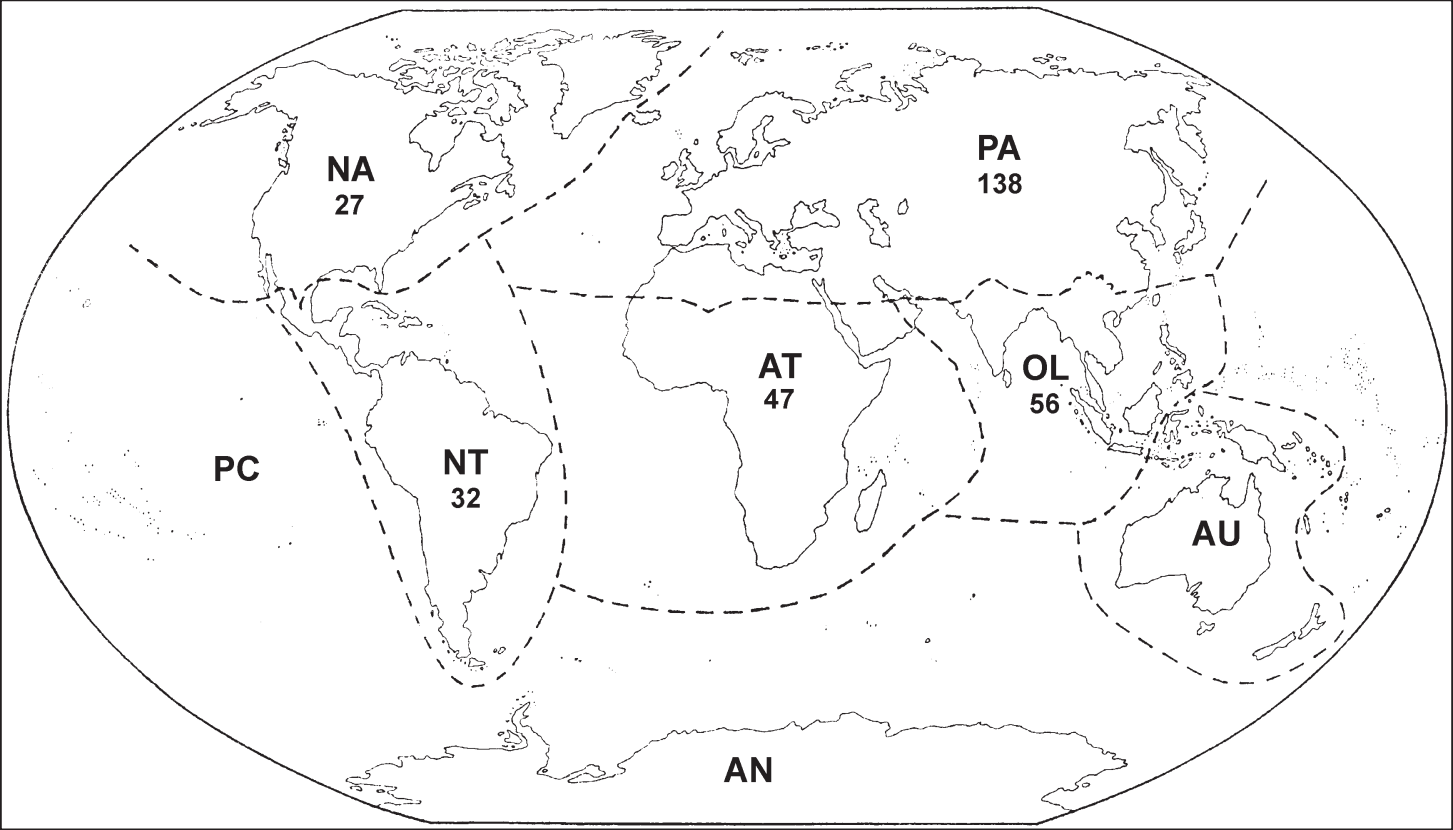
Distribution of freshwater mite species of *Atractides* per biogeographical provinces: PA–Palaearctic; NA–Nearctic; NT–Neotropical; AT–Afrotropical; OL–Oriental; AU–Australasian; PC–Pacific Oceanic Islands; AN–Antarctic. Biogeographical provinces are given according to Balian et al. (2008), modified to include northern Oman in the Oriental, and southern Oman in the Afrotropical region (see Smit and Pešić 2010 for discussion on the zoogeographical position of Oman).

To get an overview of the biodiversity of the genus worldwide, we examined numerous papers, Gerecke (2003) and the website www.watermite.org (viewed on January 23, 2011). The total number of *Atractides* species worldwide tallies exactly 297 species, including the new species described in this paper. Most species are known from the northern Hemisphere: 138 are described from the Palaearctic, most of these from the Western Palaearctic and the countries surrounding the Mediterranean Sea (Gerecke 2003). In the Oriental region 56 species have been found, 47 species have been found in the Afrotropical region, including the new species from Ethiopia, while 32 and 27 species are described from the Neotropical and Neartic region, respectively (Fig. 4).

### b) The biodiversity of Atractides in Afrotropical region

Thus far, 47 species and 3 subspecies have been recorded from the Afrotropical region, with large differences in the state of knowledge of different regions. The number of known species ranges from 14 from Kenya, 13 from South Africa and 9 from Liberia (Viets 1970; Jansen van Rensburg 1974). Conversely, only two species, *Atractides contemptus* (Lundblad, 1951) and *Atractides ethiopiensis* sp. n. are known from Ethiopia (Motaş and Tanasachi 1968, present paper).

The Afrotropical members of *Atractides* belong to the following subgenera *Atractides* Koch, 1837 *s.s*., *Megabates* K. Viets, 1924, *Tympanomegapus* Thor, 1923 and *Polymegapus* K. Viets, 1926. However, the older data (Viets 1970; Jansen van Rensburg 1974), as well as the most recent catalogue of water mites (Viets 1987) assigned *Tympanomegapus* and triacetabulate members of *Polymegapus* to *Atractides* Koch, 1837 *s.s*.

A critical analysis of the Afrotropical *Atractides* species, with the aid of the diagnoses and the revised key presented in Gerecke (2003), shows that nine species (*i.e.*, *Atractides harrisoni* K.O. Viets, 1971, *Atractides levipapis* Bader, 1968, *Atractides neotestudo* Cook, 1966, *Atractides paratestudo* Cook, 1966, *Atractides pseudotestudo* Cook, 1966, *Atractides scutifer* (Lundblad, 1951), *Atractides subtestudo* Cook, 1966, *Atractides testudo* Cook, 1966 and *Atractides tuberipalpis* (K. Viets 1913)) should be assigned to *Tympanomegapus*, while four species (*i.e.*, *Atractides abruptus* Cook, 1966, *Atractides congoensis* K.O. Viets & Böttger, 1972, A*. kuhnei* (K. Viets, 1911) and *Atractides multiporus* Cook, 1966) should be assigned to the subgenus *Polymegapus*. The subgeneric position of *Atractides lautus* K.O. Viets & Böttger, 1972 is unclear. Due to P-1 by far longer than high and centrally narrowed, this species agrees with members of *Tympanomegapus*, but differs in a stout cheliceral basal segment (L/H 2.4, calculated from Viets and Böttger 1972) and a remarkably long claw (Viets and Böttger 1972).

The subgenus *Megabates* K. Viets, 1924, includes two Afrotropical species, i.e., *Atractides rectipes* (K. Viets, 1924) and *Atractides longicoxalis* (Cook, 1974). According to Gerecke (2003), *Megabates* is most probably a synonym of *Atractides*.

The other 35 species and subspecies are assigned to *Atractides* *s. s.* According to our present state of knowledge, slightly more than a half (54%) of these species are known from both sexes, i.e., *Atractides comorosensis* Smit & Pešić, 2010, *Atractides contemptus* (Lundblad, 1951), *Atractides coriacellus* K. Viets, 1956, *Atractides damkoehleri* (K. Viets, 1916), *Atractides ethiopiensis* sp. n., *Atractides falcipes* (Walter & Bader, 1952), *Atractides kilimandjaricus* Lundblad, 1952, *Atractides latisetus* (K. Viets, 1916), *Atractides linearis* (Lundblad, 1927), *Atractides lundbladi lundbladi* (Halik, 1947), *Atractides madagascariensis* K.O. Viets, 1964, *Atractides minutissimus* (Lundblad, 1927), *Atractides processiferus* (Walter & Bader, 1952), *Atractides pusillus* (Walter & Bader, 1952), *Atractides scutelliferus* K.O. Viets, 1964, *Atractides splendidus splendidus* (Lundblad, 1927), *Atractides splendidus superbus* (Lundblad, 1927), *Atractides thoracatus* Koenike, 1898 and *Atractides valididens* (Lundblad, 1951). Three species are known from the male only, *i.e*. *Atractides africanus* (Lundblad, 1951), *Atractides baderi* K.Viets, 1956 and *Atractides invidendus* K.O.Viets, 1964, while 12 species are known from the female only, *i.e.,* *Atractides assimilis* K.O.Viets, 1964, *Atractides callosus* K.O.Viets, 1972, *Atractides exiguus* Lundblad, 1952, *Atractides immodestus* (Walter & Bader, 1952), *Atractides irangiensis* K.O. Viets & Böttger, 1972, *Atractides kuhlmanni* K.O.Viets, 1963, *Atractides lundbladi curvitarsis* K. Viets, 1955, *Atractides pulcher* K. Viets, 1956, *Atractides rostellatus* K.O.Viets, 1964, *Atractides sudafricanus* K. Viets, 1956, *Atractides tenuipes tenuipes* Lundblad, 1952 and *Atractides tenuipes ambiguus* K.O. Viets, 1971.

Three species are of doubtful status and merit attention during future taxonomical studies: *Atractides africanus* (Lundblad, 1951) – possibly a synonym of *Atractides linearis* (Lundblad, 1927) (see Viets 1964 for a discussion); *Atractides pusillus* (Walter & Bader, 1952) - rather similar to and probably a synonym of *Atractides damkoehleri* (K. Viets, 1916) (see Viets 1964 for a discussion), and *Atractides processiferus* (Walter & Bader, 1952) – there is good reason to assume that this species will be found to be synonymous with *Atractides valididens* (Lundblad, 1951), a species overlooked in the original description of *Atractides processiferus* (see: Walter and Bader 1952).

In conclusion the current knowledge of the diversity of Afrotropical *Atractides* species is far from complete. Moreover, information on the diversity of Afrotropical *Atractides* among different freshwater habitats is unbalanced, and some important habitats are poorly (e.g., springs) or completely unexplored (e.g., hyporheic interstitial). Additional field work is highly needed for an appropriate evaluation of the extant diversity.

## Supplementary Material

XML Treatment for Atractides (Atractides) ethiopiensis
